# Wild Carrot Differentiation in Europe and Selection at *DcAOX1* Gene?

**DOI:** 10.1371/journal.pone.0164872

**Published:** 2016-10-21

**Authors:** Tânia Nobre, Manuela Oliveira, Birgit Arnholdt-Schmitt

**Affiliations:** 1 EU Marie Curie Chair, ICAAM - Instituto de Ciências Agrárias e Ambientais Mediterrânicas, Universidade de Évora, Évora, Portugal; 2 Centro de Investigação em Matemática e Aplicações, Instituto de Investigação e Formação Avançada, Universidade de Évora, Évora, Portugal; Washington University, UNITED STATES

## Abstract

By definition, the domestication process leads to an overall reduction of crop genetic diversity. This lead to the current search of genomic regions in wild crop relatives (CWR), an important task for modern carrot breeding. Nowadays massive sequencing possibilities can allow for discovery of novel genetic resources in wild populations, but this quest could be aided by the use of a surrogate gene (to first identify and prioritize novel wild populations for increased sequencing effort). Alternative oxidase (AOX) gene family seems to be linked to all kinds of abiotic and biotic stress reactions in various organisms and thus have the potential to be used in the identification of CWR hotspots of environment-adapted diversity. High variability of *DcAOX1* was found in populations of wild carrot sampled across a West-European environmental gradient. Even though no direct relation was found with the analyzed climatic conditions or with physical distance, population differentiation exists and results mainly from the polymorphisms associated with *DcAOX1* exon 1 and intron 1. The relatively high number of amino acid changes and the identification of several unusually variable positions (through a likelihood ratio test), suggests that *DcAOX1* gene might be under positive selection. However, if positive selection is considered, it only acts on some specific populations (i.e. is in the form of adaptive differences in different population locations) given the observed high genetic diversity. We were able to identify two populations with higher levels of differentiation which are promising as hot spots of specific functional diversity.

## Introduction

Crop plants typically include only a portion of the genetic diversity of their wild relatives. Since genetic variation is the raw material of evolution, low genetic diversity has as direct consequence a reduction on the ability of the species to evolve in response to changes in its environment. If until recently we have perceived plant breeding as overall reducing crop genetic diversity, recent assessments have shown spatial and temporal patterns of genetic diversity losses (e.g. [1,2]). Particularly, breeding objectives have changed across time, from just yield improvement to also accommodate adaptation capacity and stress resistance under different agricultural systems and climatic conditions. This, in turn, has as consequence different selective pressures within breeding populations, and thus variable genetic diversity in released cultivars of a given crop [2]. Also, the extent of this loss of diversity depends on the population size during the domestication period, the mating system and the duration of that period, and it is not experienced equally by all genes in the genome [3]. Genetic diversity is lowered by intraspecific hybridization as well as by the selection process, which enhances genetic differentiation [4].

For many crops, like maize and cauliflower, this diversity loss due to domestication has made the plant totally dependent on humans in such a way that plant crop is no longer capable of propagating itself in nature [5]. In others however, such as in carrot (*Daucus carota* L.), the domestication process rendered more modest changes when compared to their progenitors—and they can even revert to the wild or become self-propagating weeds [5]. Even though there is clear evidence for diversification between wild and cultivated *D*. *carota* (e.g. [6,7]) and for the separation of the cultivated germplasm into two distinct groups (the Eastern–Asian and Western–European and American- gene pools [8]), widespread hybridization and introgression events have been reported (e.g. [9,10]). The outcrossing nature of carrot without any clonal propagation, associated with the fact that open-pollinated seed production was (likely) used to propagate carrot during domestication, lead to a small reduction in the genetic diversity of cultivated *vs*. wild carrot [6]. Nonetheless, Grzebelus and co-workers [11] encountered an overall higher gene diversity of wild accessions. They found private markers not present in any gene pool of cultivated carrot, which encourages the search of genomic regions potentially important for modern carrot breeding.

Close relatives of domesticated plants -crop wild relatives (CWRs)- represent a practical gene pool that can be exploited by plant breeders in the quest to address modern agricultural needs: higher productivity, climate resilience and nutritional security (reviewed in [12]). In the case of carrot, and because primary CWR are inter-fertile with the crop species, this genetic variability is easily accessible for plant improvement. To access this genetic pool, there is a need to screen wild populations. This screen can be done randomly, but can potentially be made more efficient by making use of climatic information, to help in selecting the most appropriate populations for further testing. This assumes that CWRs are adapted to their environment and thus present a set of traits involved in adaptation. Usually this means that a few genes of large effect should account for a relatively large proportion of the genetic differentiation between adapted populations together with many loci of smaller effect [13,14]. Nowadays massive sequencing possibilities can allow for discovery of novel genetic resources in wild populations, and comparison of genome variation in contrasting environments may bring new options for use of genetic variability in plant breeding for climate resilience [15]. An alternative option to massive sequencing would be the use of a surrogate gene(s) to first identify and prioritize novel wild populations for increased sequencing effort. In this case, a surrogate gene would be a proxy for the broader genome (as this is too big to allow to be considered individually for a greater number of populations) given a certain trait, for example, specificities of the environment of the adapted population. Such a surrogate gene would have to show a set of polymorphisms whose presence would link to particular environmental conditions, and indicate the presence of wider genetic variability associated with evolution of adaptation.

The adaptation to different environments involves adaptation to biotic and abiotic stresses, and the concomitant variation in CWR genes could be explored as a way to increase tolerance and hence improve long term crop productivity. Alternative oxidase (AOX) gene family seems to be linked to all kinds of abiotic and biotic stress reactions in various organisms (e.g. [16–18]) and have the potential to be used in a model towards identification of CWR hotspots of environment-adapted diversity. The choice of this gene relates to the fact that AOX is not only part of the stress response in plants, but it also plays a central role in defining the stress response [19]. By this reason, AOX was previously proposed as a source for functional markers for breeding [20]. The rendering proteins are active in mitochondria, organelles of crucial importance for environmental stress perception and stress signal transduction. This gene family is present in the respiratory chains of all plants, as well as in certain fungi, protists, animals and bacteria, and it is crucially involved in the adaptive regulation of metabolism. Within the same species, individual genotypes and/or groups of genotypes can be distinguished by polymorphic AOX gene family sequences (e.g. [20–23]) and this is also true for carrot (e.g. [24–27]). In carrot, this multigene family is encoded by three genes distributed in two discrete gene subfamilies–*DcAOX1* and *DcAOX2* (a and b). Generally, and while AOX1 is induced by stress stimuli, AOX2 is referred as constitutive or developmental. Even though this paradigm begins to be challenged [28–30] the current view is still that the subfamilies have different physiological roles. They are thus expected to have evolved under different selection pressures, being AOX1 gene subfamily likely most responsive to environmental stimuli. Campos and co-workers [31] showed that *DcAOX1* gene expression respond to different growth temperature conditions, and that this response was genotype dependent. In carrot, the complete gene shows different lengths and the less typical AOX structure of three exons interrupted by two introns, with the highest sequence variability found on intron 1 (including a hyper- variable region) followed far by exon 1 [27].

In this study we look into the variability of *DcAOX1* in populations of carrot CWR in Europe, subjected to different climatic stress and thus putatively adapted to different environments. By scanning wild crop relatives with a stress related gene, we expect to highlight hot spots of specific functional diversity.

## Material and Methods

### Plant material

Sampling was performed following an environmental gradient across Europe. This gradient accommodated sampling points with deviating climatic conditions, such as in Sierra de Guadarrama (considerable temperature changes between summer and winter and a very dry summer; wild carrots could not be found above 1100 m) or in central Pyrenees and the French Massif Central (with a cold continental climate at equivalent height). In general, the sampling was made on easily accessible non-cultivated fields (thus close to a road), with altitudes ranging from the referred 1100 m to sea level. In total, 13 sites were sampled (Table 1) by collecting wild carrot roots at each location. The samples were dried in silica gel and stored at -80°C. Due to the hard and woody characteristics of the wild carrot roots, an adaptation of the DNeasy Plant Mini Kit (Qiagen, Hilden, Germany) standard extraction protocol was used to extract the DNA: 1) extra initial grinding step (with liquid nitrogen and using a tissue grinder) to further pulverize the hard root tissues; 2) addition of polyvinylpyrrolidone (PVP, 10 000 mol wt at 3%) to the extraction buffer to remove phenolic and other compounds that can inhibit PCR and 3) lysis was performed overnight at 60 rpm. DNA concentration of all samples was determined with the NanoDrop-2000C spectrophotometer.

**Table 1:** Sampling locations and geographic coordinates of collection sites.

Population code	Name of closest location	Country code	Coordinates		
			Longitude	Latitude	Altitude
1	Guadalupe	PT	38.5825	-7.9980	310
3	Sant Carles de la Rapita	SP	40.6267	0.6529	0
4	Rostock	DE	54.0779	12.1126	24
5	Romangordo	SP	39.7795	-5.6966	327
6	Rascafria	SP	40.8840	-3.8867	1190
7	Torla-Huerdesa	SP	42.6511	-0.1380	1279
8	Gruissan	FR	43.1010	3.1201	84
9	Saint-Privat-d'Allier	FR	45.0012	3.6850	1009
10	Agencourt	FR	47.1324	4.9836	281
11	Kapellen	LU	49.6401	6.0184	362
12	Nijkerk	NL	52.2154	5.4779	2
13	Capbreton	FR	43.6307	-1.4322	64
14	Torquemada	SP	42.0364	-4.2824	785

### Data collection

The available climatic data of the last 15 years was collected for the closest by station of the sampling points (http://globalweather.tamu.edu/). Month averages were obtained to have a workable characterization of the long-term climatic conditions at the sampling locations. A Principal Component Analysis (PCA) was used to define patterns of climatic conditions of the sampling locations. Data was grouped into clusters for data summarization, through an Agglomerative Hierarchical Clustering (Ward agglomeration method, on Euclidian distances for five classes). These analyses were performed using XLSTAT-ecology, an add-on to MS Excel^®^.

For the isolation of AOX1 amplicon in wild carrots, specific primers were designed based on the already available cDNA sequence at the NCBI GenBank (EU286573.2). A nested PCR approach was selected. For the first reaction, the primers used were located at the beginning of exon 1 (*DcAOX1* _24Fw or *DcAOX1* _94Fw) as forward and at the end of exon 3 as reverse (*DcAOX1* _1032Rev; more details in [27]). A standard 25 ul reaction, at 2.5 mM of magnesium and BSA at 0.4 ug/ul, was run with an annealing temperature of 55°C. The PCR product was then diluted in 1:50 and used as template for the second reaction. The second reaction was performed with the same forward primer and the degenerated primer P2 [32], in a 25 ul reaction at 1.5 mM of magnesium. Annealing temperature was 60°C. Amplicons were purified from the agarose gel with GFX PCR DNA and Gel Band Purification Kit, directly cloned into pGEM^®^-T Easy vector (Promega, Madison, WI, USA), transformed into bacterial strainJM109 (Promega, USA) and bacterial colonies were tested using T7 and SP6 primers. Sequence was done from the PCR product, in sense and antisense strands. Because carrot is an outcrossing species, a minimum of four plants per population and two clones per plant were sequenced (S1 Table). For the amplification of D28n marker (for taxonomic certainty), we followed the procedure described by Spooner and co-workers [33], in a standard 25 ul reaction at 1.5 mM of magnesium and an annealing temperature of 55°C. Sequence was done directly from the PCR product, in sense and antisense strands.

### Phylogenetic analyses

Sequence visualization was performed in CLC Main Workbench vs 6.8.1 software and, for AOX1 amplicon, exons and intron regions were identified on the sequences and aligned separately. The alignment of segments of the exon regions was relatively straightforward, with few insertions/deletions needing to be inferred. For the intron region an iterative refinement method, which accounts for larger gaps in the sequences (E-INS-i) as implemented in the program MAFFT [34], was used to align the segment. Alignment of D28n amplicon was made under the option L-INS-I (iterative refinement method incorporating local pairwise alignments; gap opening penalty: 1.5 and gap extension penalty 0.14; 1PAM/k ¼ 2 scoring matrix for nucleotide sequences). The alignment of D28n fragment was straightforward, as no insertions/deletions had to be inferred. The optimal models of evolution were tested independently of the sequence region in MrModeltest 2.2 [35] and selection took place on the basis of the BIC scores (Bayesian Information Criterion; the lowest the value the better the substitution pattern).

Bayesian inference was conducted using MrBayes version 3.0 [36,37]. The default settings of MrBayes were used and with MCMC (considering 100 000 generations) runs being repeated three times as a safeguard against spurious results. The first 1 000 trees were discarded as burn-in, and the remaining trees were used to calculate a majority rule consensus tree. Stationarity was confirmed by analysis of the log-likelihoods and the consistency between runs. For AOX1, the same analysis was also done considering the full fragment, the exons alone and also considering the full fragment excluding the intron insertions suspected to be the result of introgression events (S1, S2 and S3 Figs).

### AOX1 variability

The AOX1 sequences alignment was used to locate polymorphic positions and ambiguities encountered were, whenever possible, resolved by resequencing. Summary statistics and tests of neutrality were calculated with DnaSPv.4.0 [38] on the basis of the number of segregating sites (since we observed three different bases per site in some populations, we also performed the analyses on the basis of the total number of mutations (h) and obtained qualitatively similar results; data not shown). Tajima’s D statistics compares the average number of pairwise differences with the number of segregating sites [39]. Over the all sequenced AOX1 fragment, linkage disequilibrium was measured using the ZnS statistic (the squared allele frequency correlation r^2^, [40]) on the basis of the parsimony informative sites. Statistical significance for ZnS and Tajima’s D was assessed by coalescent simulations with 10 000 replicates as implemented in DnaSP v. 4.0, conducted considering all segregating sites and an intermediate level of recombination [38]. In addition, for the coding regions of *DcAOX1*, tests for positive selection were performed using the maximum likelihood methods implemented in the CODEML program of PAML [41]. The dN/dS ratio (ω) was calculated using models M0 (one-ratio) and M3 (discrete), and M1a (nearly neutral) and M2a (positive selection). The relevant likelihood ratio tests were performed to access significance: M0-M3 tests for variable ω among sites and M1a-M2a tests positive selection.

We determined pairwise FST values among wild *D*. *carota* populations using Arlequin3.5.22 [42]; levels of significance were assessed on the basis of 10 000 permutations. The significance of isolation-by-distance was tested with a Mantel test.

## Results

**Fig 1:**
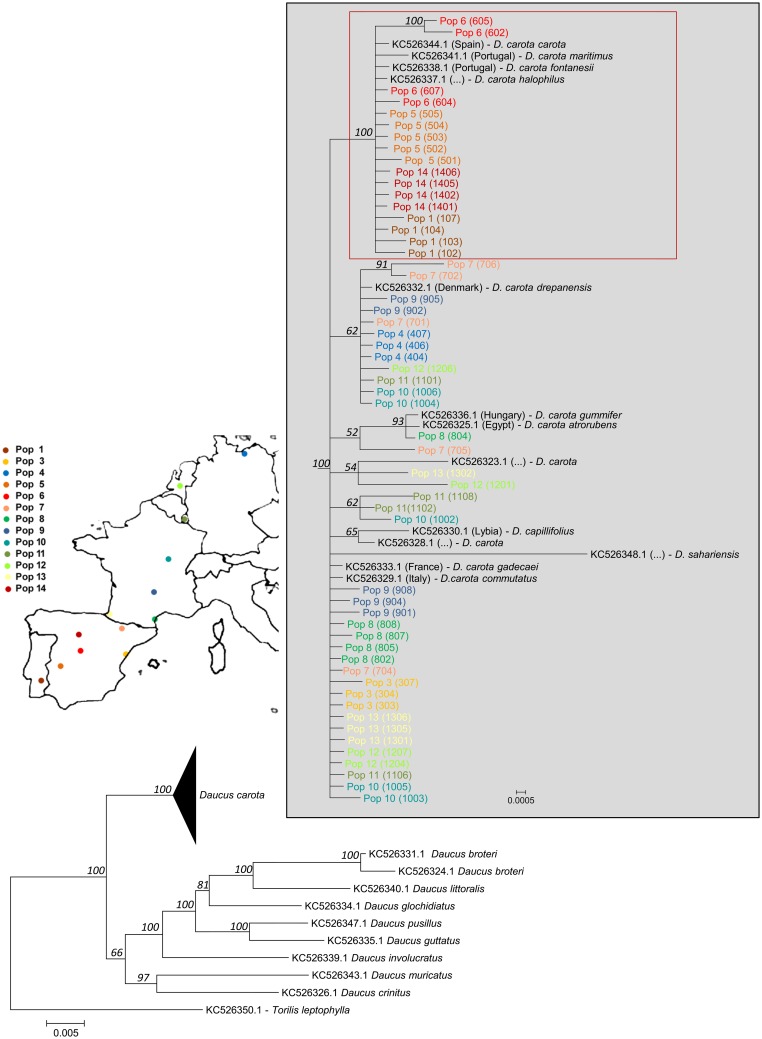
Reconstructed phylogeny based on de conserved orthologue marker D28n [33]. The phylogeny corresponds to the majority rule consensus tree of trees sampled in a Bayesian analysis (K2P+G as substitution model). *Torilis leptophylla* was used as outgroup. The numbers above the branches refer to the Bayesian posterior probability of the nodes (more than 50%) derived from 19500 Markov chain Monte Carlo-sampled trees. The red box encompasses a clade of western Iberian populations.

**Fig 2:**
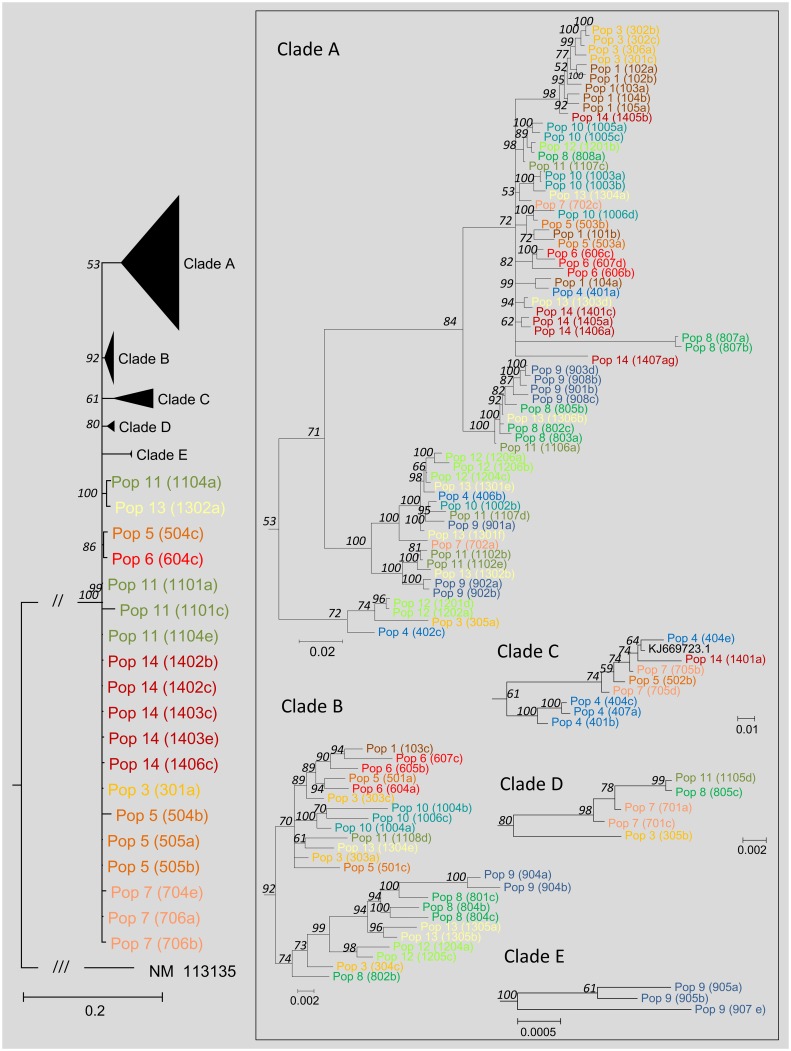
Reconstructed phylogeny based on the AOX1 fragment (2353 bp in the alignment). DNA models of evolution were tested independently of the sequence region, and selection took place on the basis of the BIC scores (Bayesian Information Criterion; the lowest the value the better the substitution pattern): Exon1—K2+G+I; Intron 1—GTR+G and Exon 2—GTR+G. The phylogeny corresponds to the majority rule consensus tree of trees sampled in a Bayesian analysis. *Arabidopsis thaliana* was used as outgroup. The numbers above the branches refer to the Bayesian posterior probability of the nodes (more than 50%) derived from 19500 Markov chain Monte Carlo-sampled trees.

**Fig 3:**
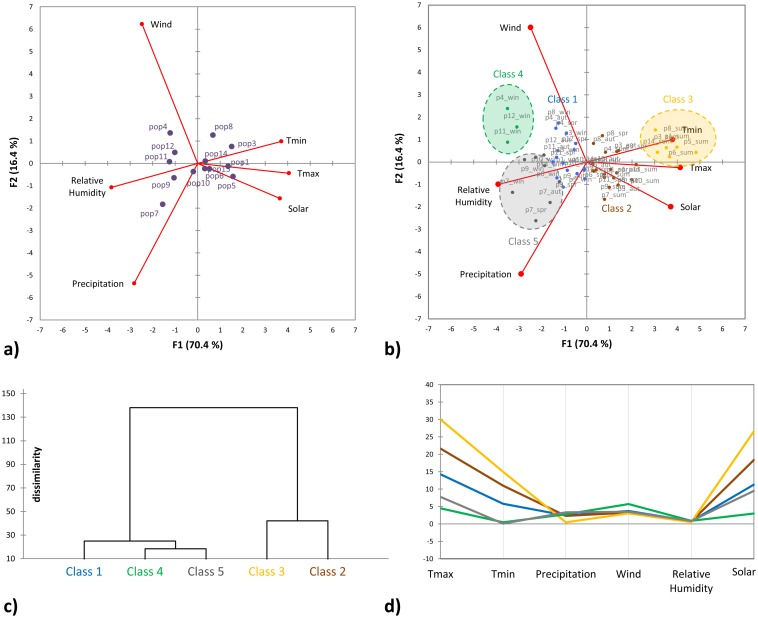
Sampling locations analysis based on average monthly weather data. A plot on the two main components of a Principal Component Analysis (86.9%): a) variables yearly averaged per population as additional category data; b) variables grouped by meteorological season. Agglomerative Hierarchical Clustering analysis: c) euclidian distance dendogram for 5 classes; d) profile of the classses. tmin, minimum temperature (°C); tmax, maximum temperature (°C); precipitation (mm); relative humidity (%); wind (m/s); solar (MJ/m^2^).

Based on the D28n phylogeny, all plants were confirmed belonging to *Daucus carota*. Within *D*. *carota* the phylogeny is highly unresolved (Fig 1), with the exception of a cluster comprising the four most western Iberian populations (Fig 1, red box). The reconstructed AOX1 phylogeny (Fig 2) however, does not show any obvious population clade or relates to a clear geographical origin. Through the use of a PCA (Fig 3a and 3b), the set of climatic observations of possible correlated variables were converted into a set of values of uncorrelated variables (the principal components). The percentage of the variance explained by the two first components is of 86.8% (70,4.% for PC1 and 16,4% for PC2). The PCA representation permits distinguishing temperatures (max and min) and also solar radiation, from the variables precipitation, humidity and wind. In the components space, population 7 sampling site is differentiated from the rest and the sampling sites of populations 4, 12 and 11 are similar for the analyzed variables (grouped based mainly on winter conditions, Fig 3b). The cluster analysis into five groups (Fig 3c and 3d) conforms with the PCA, dividing the locations into two highly differentiated clusters: a) high temperatures and solar radiation -Class 2 and Class 3 (grouping populations 1, 3, 5, 6, 8 and 14 summer characteristics); b) stronger winds and higher moisture -Class 1 and Class 4 (comprising populations 4, 11 and 12 winter conditions). Even though it is clear that the sampling locations show diversified climatic conditions of temperature, precipitation, humidity, solar radiation and even wind, no relation is apparent of any of these variables to the clades formed in the phylogenetic analysis of AOX1 gene fragment.

### *DcAOX1* variability in the CWR

**Fig 4:**
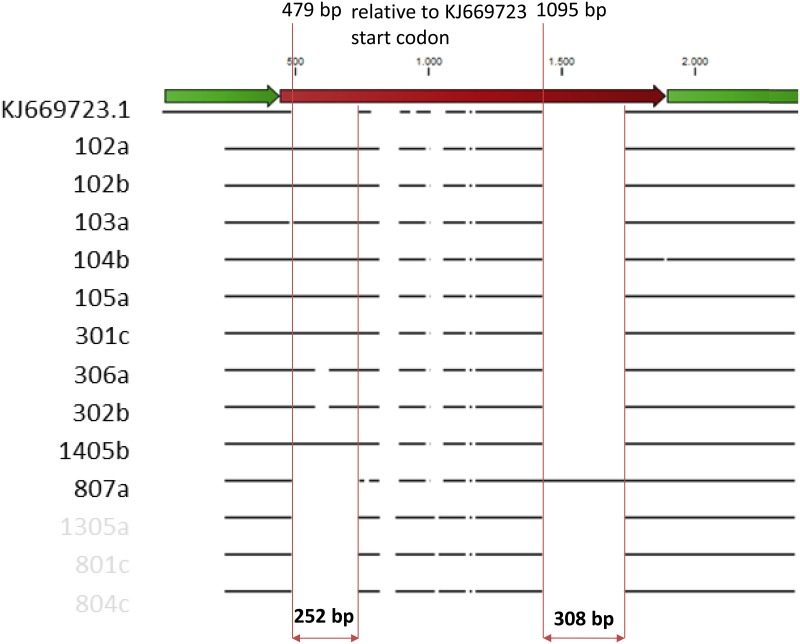
Diagram showing the long intron insertions relative to a known full DcAOX1 gene sequence (KJ669723.1).

The obtained 122 sequences were annotated for exonic regions and those were analyzed separately from the intron. Overall, the data show much higher gene diversity than initially expected: the amplicon size varies considerably due to intron 1 size, which ranged from 325 bp to 951 bp. In the current dataset, comprising a large West-European sampling of wild carrots, we found 9 haplotypes with an insertion ranging 200 bp to 252 bp in the beginning of the intron 1 (479 bp relative to KJ669723.1 start codon; Fig 4). This insertion was only present in individuals from three Iberian populations:

1. population 1–5 of the 8 haplotypes show here an insertion (I1a) which shows levels of similarity between themselves higher than 90%;
2. population 3–3 of the 8 haplotypes have here an insertion, being one of them equivalent to I1a (average similarity higher than 90%) whereas the two other haplotypes present a different insertion (I1b) but similar between themselves (99%);
3. population 14—only 1 of the 8 haplotypes present an insertion with 96% similarity with I1a.

I1a blasts primarily with *Rhizophagus intraradices* clone JGIBTPH-93C11 (AC237375, S2 Table). The deposited sequence is originated from a genome sequencing project of this arbuscular mycorrhiza fungus, where the fungus culture was root culture of carrot [43]. This means that the database hits to *R*. *intraradices* are most likely an artifact of contamination of the fungus DNA with co-cultured carrot cell DNA. All subsequent homologies found were with carrot samples (S2 Table) albeit not annotated as belonging to the AOX gene family. In only one individual, from population 8, a second insertion was found towards the end of the intron (1095 bp relative to KJ669723.1 start codon; Fig 4). This insertion of 308 bp shows high homology with genomic sequence of either *Daucus sahariensis* (KJ519787.1, KJ519789.1 and KJ519806.1) or *Daucus syrticus* (KJ519808.1 and KJ519807.1).

From exon1 the dataset is not complete and from the expected fragment of 432 sites, only 205 sites have no alignment gaps or missing data (analyzed only for 119 sequences due to absence of data on 302c, 402c and 807b). Data on the exons (number of haplotypes, polymorphic sites and diversity estimates) is summarized in Table 2. Both exon 1 and 2 exhibit high level of nucleotide diversity, showing also almost equivalent numbers of non-synonymous and synonymous sites. For haplotype and nucleotide diversity estimates (Hd and Pi), we chose to analyze synonymous and nonsynonymous sites jointly because the amount of coding sequence was limited and treating them separately would have led to estimates based on even a smaller number of sites [44].

**Table 2:** Sequence variability analyses of a *DcAOX1* fragment covering part of gene exon 1 and exon 2 in 122 wild carrot haplotypes.

	Exon 1	Exon 2
**Number of sequences**	119	122
**Number of sites (bp)**	207	479
**Total number of sites (excl. missing data)**	205	474
**Number of haplotypes**	71	94
**Polymorphic sites (S)**	46	55
Parsimony informative	23	34
**Total number of mutations**	49	56
Synonymous changes	25	31
Non-Synonymous	24	25
**Haplotype diversity (Hd)**	0.979	0.995
**Nucleotide diversity (Pi)**	0.029	0.015

Population genetics inferences are primarily based on two sources of information: the site frequency spectrum of mutations (SFS) -being Tajima’s D one of the most popular summary statistics- and the statistical association among those, that is, linkage disequilibrium (LD). Considering an intermediate level of recombination (gene recombination parameter R determined to be 18.60) the observed Tajima´s D value (-1.32) was significant (p = 0.01; [-1.19, 1.01] 95% confidence interval). The observed ZnS value (0.08) was not significant (p = 0.87; [0.04, 0.10] 95% confidence interval).

Likelihood ratio tests (LRTs) revealed that PAML models that allowed for adaptive positive selection fitted the exon 1 sequence data better than those which did not; this was, however, not true for exon 2 (Table 3) although models M3 fit the data significantly better than the null models M0. A total of 12 sites were identified as being under positive selection (ω > 1 for α = 0.05).

**Table 3:** Parameter estimates and likelihood scores under models of variable ω ratios among sites for exon1 and exon2 of the *DcAOX1* gene.

	Nested model pairs	Parameter estimates	-lnL	LRT	PSS
Exon1	M0: one ratio	ω = 0.125	3432.757	438.092 (p = 0.000)	na
	M3: discrete	ω_0_ = 0.037; ω_1_ = 1.014; ω_2_ = 5.925;Ƥ_0_ = 0.885; Ƥ_1_ = 0.100; Ƥ_2_ = 0.015	3213.711		15, 16, 30, 51, 54, 60, 63, 64, 66, 67, 68, 74
	M1a: nearly neutral	ω_0_ = 0.034; ω_1_ = 1.000 Ƥ_0_ = 0.886; Ƥ_1_ = 0.114	3236.036	44.644 (p = 0.000)	na
	M2a: positive selection	ω_0_ = 0.0346; ω_1_ = 1.000; ω_2_ = 5.907 Ƥ_0_ = 0.884; Ƥ_1_ = 0.101; Ƥ_2_ = 0.015;	3213.714		54, 66
Exon2	M0: one ratio	ω = 0.053	2444.668	11.115 (p = 0.029)	na
	M3: discrete	ω_0_ = 0.020; ω_1_ = 0.020; ω_2_ = 0.306; Ƥ_0_ = 0.239; Ƥ_1_ = 0.632; Ƥ_2_ = 0.128	2439.110		
	M1a: nearly neutral	ω_0_ = 0.036; ω_1_ = 1.000 Ƥ_0_ = 0.968; Ƥ_1_ = 0.032	2441.611	0.000 (p = 1.000)	na
	M2a: positive selection	ω_0_ = 0.036; ω_1_ = 1.000; ω_2_ = 1.000 Ƥ_0_ = 0.968; Ƥ_1_ = 0.018; Ƥ_2_ = 0.014;	2441.611		na

### Population differentiation based on AOX1

**Fig 5:**
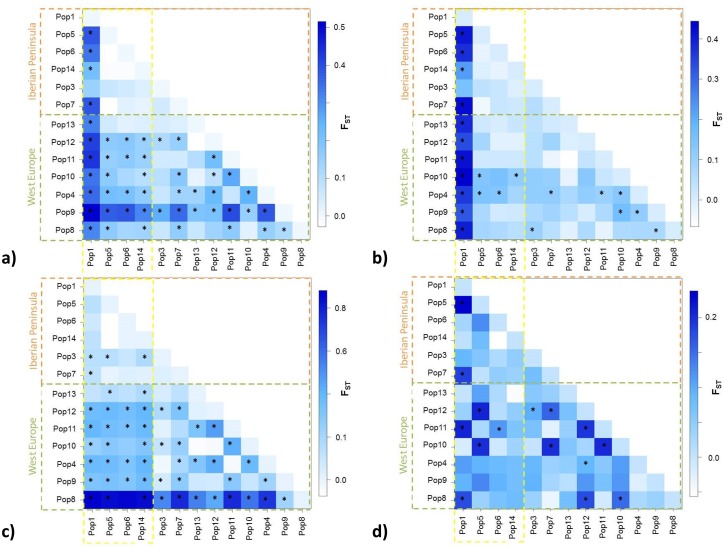
Representation of pairwise F_ST_ values among wild *D*. *carota* populations AOX1 fragment, with significance at 0.05 being highlighted with a *; a) complete fragment; b) intron1 only; c) exon1; and d) exon 2. Graphs present different y scale to highlight differences within data.

Fig 5 shows population relationships as described by F_ST_ computed between pairs of populations (measure of population differentiation due to genetic structure, ranging from 0 to 1; with zero value indicating no population structuring or subdivision and one indicating that all genetic variation at the analyzed markers can be explained by population structure). It shows that variability at exon 1 (Fig 5c) is the main responsible for differentiation, except for the case of population 1 where the indel at intron 1 (presented above; Fig 5b) characterizes the population. No isolation by distance was observed (Mantel test considering the entire sequenced fragment, p = 0.31; only intron 1, p = 0.12; only exon 1, p = 0.65; only exon 2, p = 0.77).

## Discussion

*Daucus carota* is a highly diverse group, which certainly contributes to the poorly developed (or even lack of) barriers for interbreeding among either CWR or domesticated forms. The reconstructed phylogeny of carrot CWR (based on the conserved orthologue marker D28n) suggests a Eurosiberian (Boreal)–Mediterranean division, with the exception of the Miranda del Ebro population (population 3). This population, even though still in a Mediterranean climate (Csa, according to Köppen-Geiger climate classification system), grows in an environment close to an oceanic climate zone (Cfb) much more similar, in overall weather conditions, to other more central European locations. The reconstructed phylogeny based on the analyzed *DcAOX1* gene fragment does not seem to identify any clear clade that could directly be linked to the population origin. The observed structure in the reconstructed tree is mainly due to the intron region, as the one obtained when using the exons only is generally unresolved (S2 Fig).

There is a lack of structure among the *DcAOX1* genetic pool within the Iberian Peninsula (Fig 4), and these populations seem to be differentiated from the ones of the rest of the Western Europe, consistent with the information obtained based on the D28n marker. The overall level of population differentiation is relatively low, but population 9 (Saint-Privat-d’Allier, France) and population 1 (Guadalupe, Portugal) are highlighted as potentially interesting in terms of differentiation, which results mainly from the polymorphisms associated with exon 1 and intron 1. On the contrary to what was observed with CWR of lettuce in Europe for example [45], no correlation between geographic and genetic distances was found for wild carrot based on the studied *DcAOX* gene sequences. The outcrossing nature of carrot (contrasting with the selfing habits of lettuce) increases the probability of long-distance gene flow, and hence the absence of a distance effect. The level of genetic differentiation depends on gene flow and genetic drift, so the lack of genetic differentiation among Iberian populations likely results from high rates of historical gene flow between populations and/or large effective population sizes. The intron 1 of *DcAOX1* is a lengthy variable region, including even a hyper-variable region of simple sequence repeats (SSRs) [27], and shows in the present study two large insertions (> 200 bp).

A sole wild carrot specimen from population 8 (Port-la-Nouvelle, France) showed an insertion of 308 bp at the end of intron 1 with high identity with clones of *Daucus sahariensis* and *Daucus syrticus*. Arbizu and co-workers [10] found that, even with combined molecular and morphological studies, there are particular problems in distinguishing these two species. *Daucus syrticus*, and *D*. *sahariensis* together with *D*. *gracilis*, are probably the most closely related plants to *D*. *carota* [46]. This insertion can either be vertically inherited from a common ancestor, or the result of a hybridization event between wild *D*. *carota* and *D*. *sahariensis* or *D*. *syrticus* (that even though native in North Africa could have been locally introduced in France). Remains to be tested whether the high variation at the intron, has an effect on gene expression and on the functionality of the encoded alternative oxidase protein. Introns can have large influence in the control of gene expression in plants (e.g. [47,48]). Particularly, introns at the proximity of the 5′ end of a gene are of relevance, as they can affect the binding of transcription factors [49], the process of alternative splicing [50], the coding of intronic regulatory elements [51] and also nonsense-mediated mRNA decay [52].

Nucleotide diversity at the *DcAOX1* fragment of carrot CWR was high (higher at exon 1 than exon 2), no linkage disequilibrium was found and the significant negative value of Tajima’s D suggests either a recent selective sweep (or linkage to it) or a recent population expansion following a bottleneck (the current data, based on one gene only, does not allow to distinguish between both events).

The likelihood ratio test identified several positions at exon 1 which are unusually variable (indication of positive selection), and a relatively high number of amino acid changes is observed, together indicating that *DcAOX1* gene might be under positive selection. However, the high genetic diversity, including several indels, suggests that, if there is positive selection, it only acts on some specific populations (i.e. is in the form of adaptive differences in different population locations). The hypothesis that balanced selection is driving the high genetic diversity at *DcAOX1* gene cannot be excluded.

Additionally, the observed high diversity suggests that the *DcAOX1* gene is not present in a single copy. The adaptive role of copy number variation (CNV) is suspected to be of high relevance, and for specific genes has been linked to important traits such as flowering time, plant height and resistance to biotic and abiotic stress (reviewed in [53]). Knowledge on the extent of CNV of the *DcAOX1* gene in natural populations will elucidate on its adaptive role.

Exon 1 in the *DcAOX1* gene seems to result from a fusion of two exons (via loss of an intron; [31]) and this intron loss might have been adaptive. It has been suggested the existence of high selection force against introns in rapidly regulated genes, with the rationality that an intron-less allele will produce its protein product more rapidly than a corresponding intron-containing allele because splicing is relatively slow compared with transcription [54]. Nonetheless, intron loss-gain seems to be a dynamic process difficult to generalize, as the importance of several introns for the functions of the respective gene might imply that, in some cases, intron insertion is favored by natural selection so that evolutionary conserved genes may accumulate introns [55].

It has been proposed that positive selection promotes the functional divergence of gene family members encoding enzymes involved in secondary metabolism as its products are thought to be a response to challenges imposed by the environment ([e.g. [55,56,57]). In plants, the AOX is present as a small multigene family in individual species with the overall proposed role in regulation of growth rate homeostasis under various environmental conditions that a plant undergoes. Umbach and co-workers [58] showed that alterations in the AOX pathway provoked changes that were largely chloroplast and carbohydrate metabolism related, and not only moderating ROS, thus contributing to the accumulation of secondary metabolites [59]. Therefore, the signatures suggesting local positive selection, the indications of high CNV and the *a priori* knowledge of the AOX1 gene involvement in homeostasis under stress conditions, calls for further characterization.

If, with the present data, AOX does not seem to work as a surrogate for diversity directly linked to the climatic conditions analyzed, we were able to clearly identify two populations with higher levels of differentiation which are promising as hot spots of specific functional diversity. These two populations are thus good targets for a wider approach, either considering several candidate genes or a genome-wide approach, towards the identification of novel genetic resources relevant for modern carrot breeding. Enlarging this study to a wider geo-climatic region, including the center of domestication of carrot, has the potential of identifying further genetic diversity hotspots, particularly if combined with a more fine scale environmental analysis.

## Supporting Information

S1 Fig Reconstructed phylogeny based on a AOX1 fragment. The phylogeny corresponds to the majority rule consensus tree of trees sampled in a Bayesian analysis. Two insertions at the intron were removed. Arabidopsis thaliana was used as outgroup. The numbers above the branches refer to the Bayesian posterior probability of the nodes (more than 50%) derived from 19500 Markov chain Monte Carlo-sampled trees.
(TIF)

S2 Fig Reconstructed phylogeny based on a AOX1 fragment. The phylogeny corresponds to the majority rule consensus tree of trees sampled in a Bayesian analysis. Only fragments in exons were considered. Arabidopsis thaliana was used as outgroup. The numbers above the branches refer to the Bayesian posterior probability of the nodes (more than 50%) derived from 19500 Markov chain Monte Carlo-sampled trees.
(TIF)

S3 Fig Reconstructed phylogeny based on a AOX1 fragment. The phylogeny corresponds to the majority rule consensus tree of trees sampled in a Bayesian analysis. Only intron 1 was considered. Arabidopsis thaliana was used as outgroup. The numbers above the branches refer to the Bayesian posterior probability of the nodes (more than 50%) derived from 19500 Markov chain Monte Carlo-sampled trees.
(TIF)

S1 Table Sample locations, geographic coordinates, populations and individual plants codes. (PDF)

S2 Table Intron 1 insertions and homologies according to NCBI. (PDF)
