# Supplementary figures and images for: Wild Carrot Differentiation in Europe and Selection at DcAOX1 Gene?
Source: PLoS One. 2016 Oct 21;11(10):e0164872. doi: 10.1371/journal.pone.0164872 (PMC5074564; doi:10.1371/journal.pone.0164872)

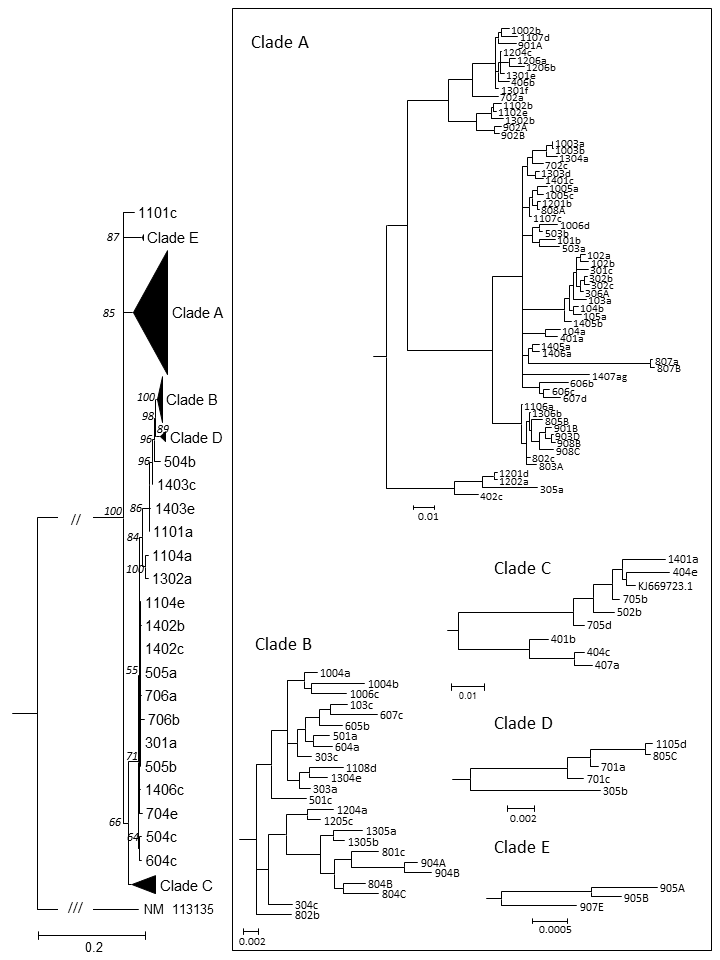

Supplement: S1 Fig — The phylogeny corresponds to the majority rule consensus tree of trees sampled in a Bayesian analysis. Two insertions at the intron were removed. Arabidopsis thaliana was used as outgroup. The numbers above the branches refer to the Bayesian posterior probability of the nodes (more than 50%) derived from 19500 Markov chain Monte Carlo-sampled trees. (TIF) [file pone.0164872.s001.TIF]

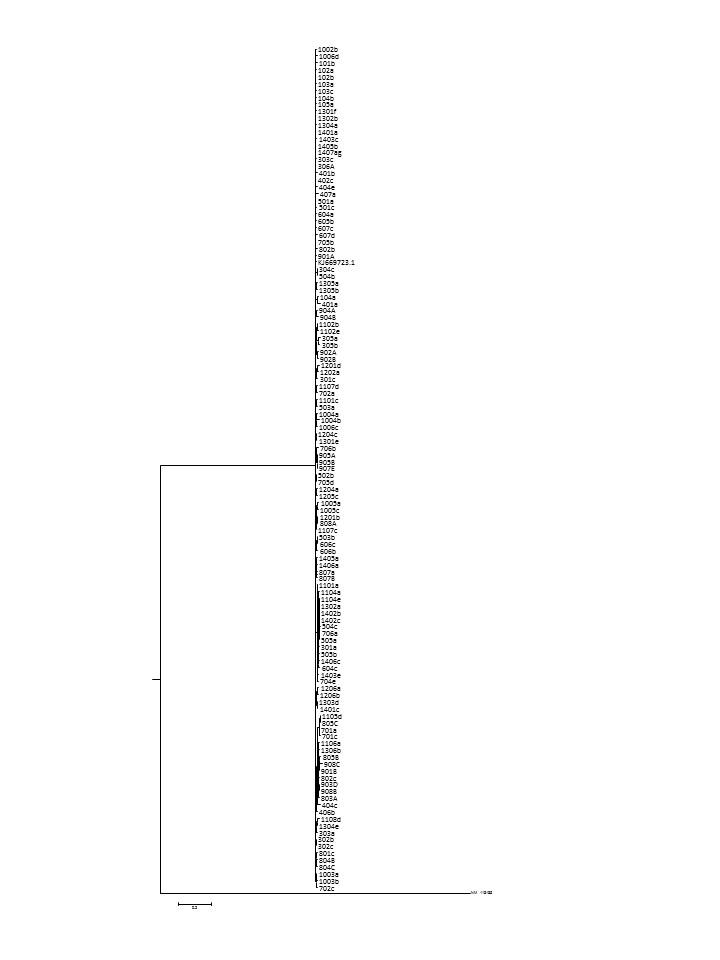

Supplement: S2 Fig — The phylogeny corresponds to the majority rule consensus tree of trees sampled in a Bayesian analysis. Only fragments in exons were considered. Arabidopsis thaliana was used as outgroup. The numbers above the branches refer to the Bayesian posterior probability of the nodes (more than 50%) derived from 19500 Markov chain Monte Carlo-sampled trees. (TIF) [file pone.0164872.s002.TIF]

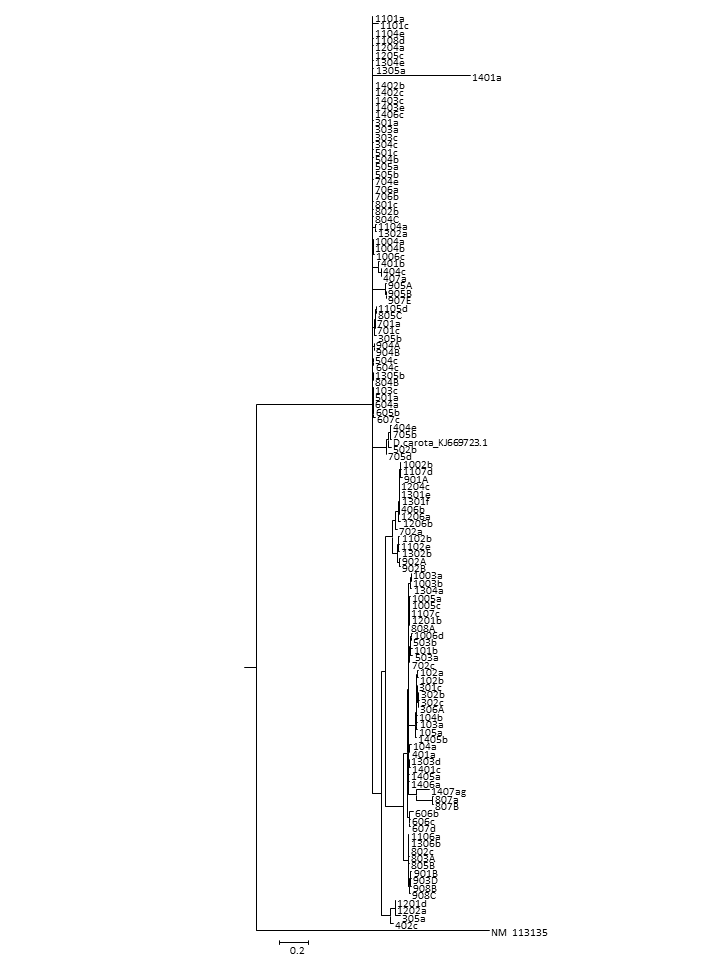

Supplement: S3 Fig — The phylogeny corresponds to the majority rule consensus tree of trees sampled in a Bayesian analysis. Only intron 1 was considered. Arabidopsis thaliana was used as outgroup. The numbers above the branches refer to the Bayesian posterior probability of the nodes (more than 50%) derived from 19500 Markov chain Monte Carlo-sampled trees. (TIF) [file pone.0164872.s003.TIF]
