# Supplementary material for: Wild Carrot Differentiation in Europe and Selection at DcAOX1 Gene?
Source: PLoS One. 2016 Oct 21;11(10):e0164872. doi: 10.1371/journal.pone.0164872 (PMC5074564; doi:10.1371/journal.pone.0164872)
Supplement: S1 Table — (PDF) [file pone.0164872.s004.pdf]

Table S1: Sampling locations, geographic coordinates of collection sites and codes of individual plants used

Population code	Name of closest location	Country code	Coordinates			Codes of individual plants	
			Longitude	Latitude	Altitude	D28n	AOX1
1	Guadalupe	PT	38.5825	-7.9980	310	102; 103; 104; 107	101; 102; 103; 104; 105
3	Sant Carles de la Rapita	SP	40.6267	0.6529	0	303; 304; 307	301; 302; 303; 304; 305; 306
4	Rostock	G	54.0779	12.1126	24	404; 406; 407	401; 402; 404; 406; 407
5	Romangordo	SP	39.7795	-5.6966	327	501; 502; 503; 504; 505	501; 502; 503; 504; 505
6	Rascafria	SP	40.8840	-3.8867	1190	602; 604; 605; 607	604; 605; 606; 607
7	Torla-Huerdesa	SP	42.6511	-0.1380	1279	701; 702; 705; 706	701; 702; 704; 705; 706
8	Gruissan	F	43.1010	3.1201	84	802; 804; 805; 807; 808	801; 802; 803; 804; 805; 807; 808
9	Saint-Privat-d'Allier	F	45.0012	3.6850	1009	901; 902; 904; 905; 908	901; 902; 903; 904; 905; 907; 908
10	Agencourt	F	47.1324	4.9836	281	1003; 1004; 1005; 1006	1002; 1003; 1004; 1005; 1006
11	Kapellen	L	49.6401	6.0184	362	1101; 1102; 1106; 1108	1101; 1102; 1104; 1105; 1106; 1107; 1108
12	Nijkerk	NL	52.2154	5.4779	2	1201; 1204; 1206; 1207	1201; 1202; 1204; 1205; 1206
13	Capbreton	F	43.6307	-1.4322	64	1301; 1302; 1305; 1306	1301; 1302; 1303; 1304; 1305; 1306
14	Torquemada	SP	42.0364	-4.2824	785	1401; 1402; 1405; 1406	1401; 1402; 1403; 1405; 1406
