# Supplementary material for: Wild Carrot Differentiation in Europe and Selection at DcAOX1 Gene?
Source: PLoS One. 2016 Oct 21;11(10):e0164872. doi: 10.1371/journal.pone.0164872 (PMC5074564; doi:10.1371/journal.pone.0164872)
Supplement: S2 Table — (PDF) [file pone.0164872.s005.pdf]

Table 1: Intron 1 insertions and homologies according to NCBI

Query	bp	# of hits	Lowest E-value	Greatest bit score	Description	Greatest hit length	Description
**102b (I1a)**	247	55	3E-39	172.61	AC237375	194	KJ516252
**104b (I1a)**	252	53	3E-44	188.84	AC237375	194	KJ516252
**102a (I1a)**	247	51	5E-36	161.79	AC237375	194	KJ516252
**103a (I1a)**	238	53	6E-47	197.85	AC237375	187	KJ516252
**105a (I1a)**	248	52	6E-47	197.85	AC237375	188	FJ147964
**301c (I1a)**	252	85	2E-48	203.26	AC237375	194	KJ516252
**1405b (I1a)**	252	85	8E-46	194.25	AC237375	194	KJ516252
**302b (I1b)**	200	108	9E-25	123.92	AC237375	94	KJ516244
**306a (I1b)**	200	105	1E-23	120.31	AC237375	94	KJ516244

AC237375: *Rhizophagus intraradices* clone JGIBTPH-93C11, complete sequence; KJ516252: *Daucus littoralis* clone lit295857 anonymous marker DC59353 genomic sequence; FJ147964: *Daucus carota* subsp. *sativus* clone BAC C126B08 genomic sequence; KJ516244: *Daucus guttatus* clone gut279763 anonymous marker DC59353 genomic sequence.
